# Supplementary material for: The self prefers itself? Self-referential versus parental standards in face attractiveness
Source: PeerJ. 2014 Sep 25;2:e595. doi: 10.7717/peerj.595 (PMC4178458; doi:10.7717/peerj.595)
Supplement: Supplemental Information 1 [file peerj-02-595-s001.doc]

**Consent Form**

I hereby agree that I will participate in the research project *Face Perception*.

My signature indicates that I have received written and oral information about the project, and participates voluntarily.

My consent includes that I will view images of faces and rank/order the images according to the faces’ attractiveness.

I further agree to answer a questionnaire about my upbringing.

I may at any time resign from the project, without stating reasons, requiring that all data related to my investigation will be deleted. Only project staff at the Department of Psychology will be able to gain access to identifiable data regarding my participation in the survey. Information about me will in unidentified form be retained for project completion.

_____________________________________ __________ _____/_____

Name Place Date

(Participant)

I confirm that I have given information about the study:

_____________________________________ __________ _____/_____

Name Place Date

(Project leader)
