# Supplementary figures and images for: The self prefers itself? Self-referential versus parental standards in face attractiveness
Source: PeerJ. 2014 Sep 25;2:e595. doi: 10.7717/peerj.595 (PMC4178458; doi:10.7717/peerj.595)

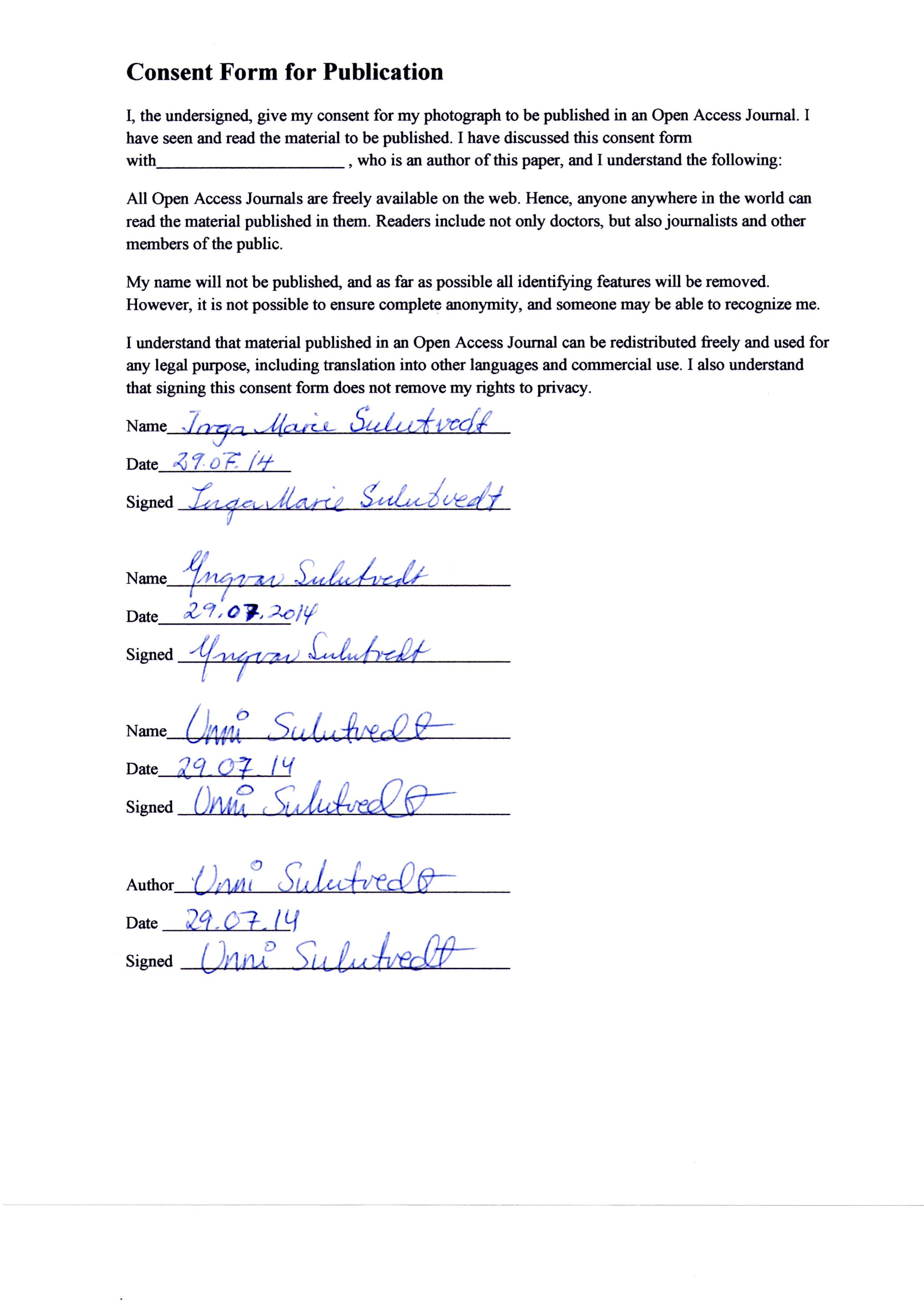

Supplement: Supplemental Information 2 [file peerj-02-595-s002.jpg]
